# The Perception of the Müller-Lyer Visual Illusion in Schizophrenics and Non-human Primates: A Translational Approach

**DOI:** 10.3389/fnbeh.2021.641776

**Published:** 2021-05-28

**Authors:** Ana Luísa Lamounier Costa, Ronaldo Coelho Silva, Pedro H. Coelho-Cordeiro, Fernando Silva da Silveira, Marilia Barros, Fabio Viegas Caixeta, Rafael S. Maior

**Affiliations:** ^1^Primate Center, Institute of Biology, University of Brasília, Brasilia, Brazil; ^2^Laboratory of Neuroscience and Behavior, Department of Physiological Sciences, Institute of Biological Sciences, University of Brasília, Brasilia, Brazil; ^3^Department of Pharmacy, School of Health Sciences, University of Brasilia, Brasilia, Brazil

**Keywords:** geometric illusion, glutamate model for schizophrenia, sensory integration, non-human primate, schizophrenia

## Abstract

The Müller-Lyer Illusion (MLI) has been suggested as a potential marker for the perceptual impairments observed in schizophrenia patients. Along with some positive symptoms, these deficits are not easily modeled in rodent experiments, and novel animal models are warranted. Previously, MK-801 was shown to reduce susceptibility to MLI in monkeys, raising the prospects of an effective perception-based model. Here, we evaluate the translational feasibility of the MLI task under NMDA receptor blockage as a primate model for schizophrenia. In Experiment 1, eight capuchin monkeys (*Sapajus* spp.) were trained on a touchscreen MLI task. Upon reaching the learning criteria, the monkeys were given ketamine (0.3 mg/kg; i.m.) or saline on four consecutive days and then retested on the MLI task. In Experiment 2, eight chronic schizophrenia patients (and eight matching controls) were tested on the Brentano version of the MLI. Under saline treatment, monkeys were susceptible to MLI, similarly to healthy human participants. Repeated ketamine administrations, however, failed to improve their performance as previous results with MK-801 had shown. Schizophrenic patients, on the other hand, showed a higher susceptibility to MLI when compared to healthy controls. In light of the present and previous studies, the MLI task shows consistent results across monkeys and humans. In spite of potentially being an interesting translational model of schizophrenia, the MLI task warrants further refinement in non-human primates and a broader sample of schizophrenia subtypes.

## Introduction

In spite of being consistently associated with schizophrenia, sensory and perceptual deficits are not considered as core symptoms nor are they used to classify its subtypes. The heterogeneity of the spectrum is mostly defined by the presence of positive or negative symptoms (Khoury et al., 2017). Animal and clinical research literature, on the other hand, has given considerable attention to these impairments and their potential as biomarkers in patients, particularly in the case of sensory gating (e.g., Swerdlow et al., 2006) and visual illusions (e.g., Pessoa et al., 2008; King et al., 2017). Susceptibility to the Müller-Lyer illusion (MLI) is a promising strategy as it has been reported to vary among schizophrenic patients (Letourneau, 1974; Parnas et al., 2001). There are several indications that the MLI may be influenced by top-down modulation from the anterior cingulate (Qiu et al., 2008) and posterior parietal cortex (Weidner and Fink, 2007; Maddaluno et al., 2019). Indeed, these areas are subject to loss of volume or gray matter or even reduced connectivity in schizophrenia patients (Rimol et al., 2010; Roiser et al., 2013). Since these morphometrical changes may intensify as a function of time, sensitivity to the MLI may vary according to the stage of the illness (Parnas et al., 2001).

As a tool for animal model research, sensitivity to the MLI seems to be remarkably conserved across species. It has been identified in primates (Suganuma et al., 2007; Tudusciuc and Nieder, 2010), birds (Warden and Baar, 1929; Winslow, 1933; Pepperberg et al., 2008), and even fish species (Sovrano et al., 2016). Also, the degree of sensitivity, measured in visual angle, seems to be quite similar between naïve monkeys and healthy humans (Tudusciuc and Nieder, 2010). These reports indicate that the MLI may be an illusory byproduct stemming from fundamental features of visual organization and/or processing. It also translates into a stable parameter for cross-species comparisons using similar behavioral tasks.

Few pharmacological models of schizophrenia have been used so far to investigate sensory impairments in nonhuman primates. NMDA receptor blockade by MK-801 increases prepulse inhibition in monkeys (Saletti et al., 2015, 2017), similar to results found with rodents (Gomes et al., 2014). Although sensory gating protocols are commonplace with rodents, visual impairment experiments are not quite as feasible. A translational model of these alterations would, therefore, be more suitable in nonhuman primates. Previously, we introduced a new research protocol in capuchin monkeys to test the effects of NMDA receptor blockade on their sensitivity to MLI (Jacobsen et al., 2017). At very low doses, MK-801 improved performance on the MLI task, an indication of reduced sensitivity to the illusion effect. Increased doses of MK-801, however, are liable to ataxia which would by itself impair the motor performance required on the task. This would preclude a possible dose-dependent sensitivity curve in the MLI. Here, we attempted an alternative strategy of NMDA receptor blockade by means of ketamine. As a drug with approved clinical use both in veterinary and human medicine, its profile may foster easier comparisons between human and nonhuman results. Furthermore, we tested a novel MLI task on chronic schizophrenic patients. The results are discussed in light of using the MLI task as a translational model of perceptual changes in schizophrenia.

## Materials and Methods

### Experiment 1: Effects of Ketamine on MLI Task (Capuchin monkeys)

#### Subjects

Five adult (10–20 years old) capuchin monkeys (*Sapajus* spp.) were used in this study, four females and one male, weighing between 2 and 5 kg. They were housed in pairs or triads at the Primate Center of the University of Brasilia, Brazil, with home-cages (4 m long, 2.9 m wide, and 2 m high) being provisioned with natural substrate, rope swings, and nest boxes. The animals were tested in their own home-cages under natural light and temperature conditions. They were separated from the rest of their group only during the training and test sessions (see “Procedure” section below). No head or body restrain was enforced. All subjects had prior experience with touchscreen monitors, yet none had been previously exposed to the drug tested. The capuchins had free access to food and water, except during the experimental sessions. All the procedures in the animal experiments were approved by the Animal Ethics Committee of the University of Brasilia (46077/2014) and complied with the Brazilian regulations for the scientific use of laboratory animals (Lei Arouca 11.794/2008), as well as the CONCEA/Brazil and NIH/USA guidelines for the care and use of laboratory animals.

#### Apparatus and Computer Program

A laptop (Lenovo^®^, Intel Core i7^®^, Brazil) connected to a 15 in. touchscreen monitor (Elo Touchsystems^®^, Brazil) was used for data collection. The apparatus was set up in front of the cage’s wire mesh door, about 20 cm from the animal. The area behind the monitor was covered with a black cloth to reduce visual distractions. All training and test tasks were created and run using the E-Prime 2.0 software (Psychology Software Tools Inc^®^, USA), with the subject’s response accuracy being recorded on each session.

#### Procedure

The training procedures were held 5 days a week, between 8 and 12 AM, being divided into three stages: an initial training phase; the determination of each subject’s Point of Subjective Equality (PSE) with and without arrowheads; and lastly a test stage. On all three stages, 5 mm thick straight horizontal black lines on a white background were used as stimuli. These lines were 20–105 mm in length (4–20 visual degrees), with or without arrowheads at the extremities. When present, the length of the arrowheads was 25% of the length of the respective line, forming a 45° angle for outward-pointing arrowheads and 135° for inward-pointing arrowheads. Correct and incorrect responses from the subjects on each task were immediately followed by distinct (0.5 s) buzzer tones. Each correct response was also rewarded with a raisin provided manually to the subject by one of the experimenters.

#### Training

**Figure 1:**
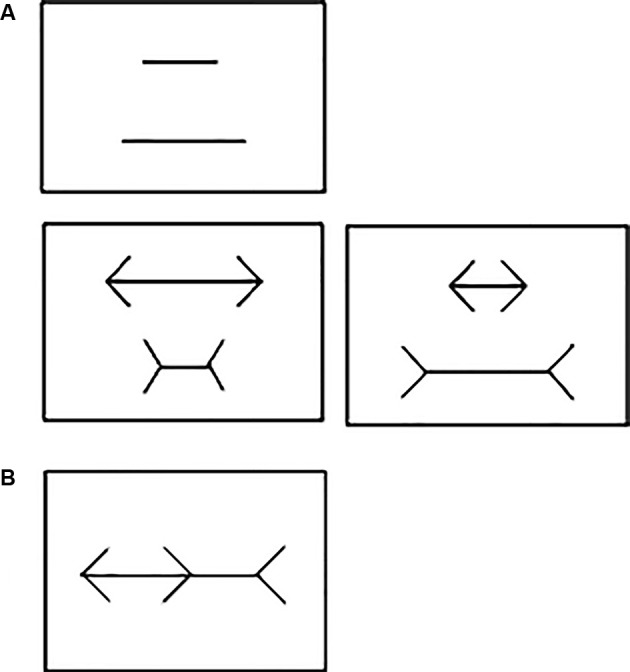
**(A)** Example of the stimuli used during training, point of subjective equality (PSE) and drug administration sessions in Experiment 1. No arrowheads (Top); neutral pair with arrowheads (Bottom left) and illusion pair with arrowheads (Bottom right). **(B)** Brentano version of the Müller-Lyer Illusion (MLI) used in Experiment 2.

Subjects were trained for several weeks to select the shortest line in each stimulus pair (with or without arrowheads) presented on the touchscreen monitor (Figures 1A,B). Subsequently, the point of subjective equality (PSE) was determined for each animal. The PSE was operationally defined as the difference whereby the subject’s performance reached chance level.

#### Point of Subjective Equality

PSE without arrowheads: we initially calculated the percentage of correct responses required per session using a binomial test with a 95% confidence interval limit (CI) of a random performance (50%) based on the total number of trials per session. As each session consisted of 60 trials (plus five “warm up” trials not used for any analysis), the upper CI was set at 63.19% per session, i.e., 38 correct responses in 60 trials. On any given session, the length difference between the two lines presented was the same on all trials (Figure 1A, top). On the subject’s first session, the pair of lines had a 50% length difference (140/70 mm). If the subject attained the calculated percentage of correct responses on this session, the difference in length between the two lines was reduced by 10 percentage points (p.p.) on the next session (e.g., 50–10 = 40%). If the criterion was not reached within the first session, the same length difference was repeated on the subsequent session. If the criterion was still not reached, the subject’s third session tested the last length difference it had achieved the upper CI minus 2 p.p. For instance, if the upper CI was attained at 50% but not at 40% difference, the next session would use a 48% (50%–2 p.p.) length difference between lines. The procedure was then repeated with successive 2 p.p. decrements at each session until the subject failed to reach the upper CI on two consecutive sessions. The last length difference in which the calculated percentage of correct responses was reached was set as the subject’s PSE without arrowheads (e.g., if it failed at a 44% difference, then “PSE without arrowheads” for this subject would be set at 46% length difference between lines).

PSE with arrowheads: In this phase, each session consisted of 60 trials: 30 with “illusion pairs” and 30 with “neutral pairs”. For the latter, the direction of the arrowheads accentuated the length difference between the two lines (Figure 1A, bottom left), whereas it decreased that of the “illusion pair” (Figure 1A, bottom right). Only “illusion pairs” were used to determine each subject’s PSE with arrowheads and as such the upper CI was set at 68.7% per session (21 correct responses in 30 trials). A given length difference was tested on four consecutive sessions, held at 24 h intervals. The first line pair tested was the 140/70 mm, corresponding to a 50% difference in length between the two lines. If the subject attained the upper CI on all four sessions, the subsequent four sessions used a pair of lines with a 10 p.p. decrease in length difference (e.g., 50–10 = 40%). If the criterion was not reached on any of these four sessions, the next four-session sequence tested the last difference the subject had achieved the upper CI minus 2 p.p. [e.g., if upper CI was attained at 50% but not at 40% difference, then the next session used 48% (50%–2 p.p.) length difference between the lines]. This procedure was then repeated on the subsequent four-session sequences, in which successive 2 p.p. decrements in length difference were tested until the subject failed to reach the upper CI on any of the sessions. The last length difference in which the upper CI was reached was set as the subject’s individual PSE with arrowheads (e.g., if failed at 44% difference, then “PSE with arrowheads” for this subject would be set at 46% length difference between lines).

#### Test

The drug challenge comprised two four-session sequences, held at 24 h intervals. The subjects were captured in their home-cages and received a vehicle or ketamine (0.3 mg/kg, i.m.) injection, respectively, 25 min prior to behavioral testing. Ketamine was dissolved in saline solution and injected in a volume of 0.5 ml/kg (this concentration was determined to reduce muscular pain and to reduce the duration of restraint). Saline was also used as the vehicle control. All sessions at this stage were carried out using the subject’s own PSE (with arrowheads) determined in the preceding stage. Each session consisted of 60 trials, 30 with “illusion pairs” and 30 with “neutral pairs”. Once again the number of correct answers was determined solely on the responses for the “illusion pairs”. “Neutral pair” trials were employed as task controls and therefore a 90% level of correct responses per session in these trials was set as a criterion to ensure that subjects were still following the task rule (i.e., choosing the shorter line) or that “illusion pairs” were not being chosen randomly.

### Experiment 2: Brentano MLI Test in Schizophrenic Patients

#### Participants

Eight chronic schizophrenia patients (all males) were inpatients recruited at the São Vicente de Paulo Hospital (Federal District, Brazil). The demographic characteristics of each patient are shown in Table 1. To be included in the study, patients had to be between 18 and 65 years of age and diagnosed with chronic/residual schizophrenia (ICD: F20.5). Exclusion criteria included: (1) any history of Traumatic Brain Injury; (2) history of a neurological disorder; and (3) current substance abuse or dependence disorder (within the past 6 months). The control group (three females; five males) was recruited to match the patient group demographics. All participants or their legal representatives were required to sign a Free and Informed Consent form prior to the start of procedures. This experiment had been previously approved by the University of Brasília Research Ethics Committee (CAAE 96510318.3.0000.0030).

**Table 1:** Demographics: schizophrenic patients (top) and controls (bottom).

Patient#	Sex	Age	Length of illness (years)	Age of diagnosis	Drugs in use	Highest school level completed	Family mental disease
1	M	59	41	18	Clozapine	Middle school	Yes
2	M	48	31	17	Clozapine + Haloperidol + Lithium	Elementary School	No
3	M	40	13	27	Clozapine + Valproic acid	High school	No
4	M	36	12	24	Clozapine + Olanzapine	Middle school	No
5	M	31	10	21	Haloperidol	Elementary school	No
6	M	37	17	20	Clozapine	High school	No
7	M	23	4	19	Clozapine	Middle school	No
8	M	37	11	26	Clozapine	Elementary school	Yes
Control#	Sex	Age	Length of illness (years)	Age of diagnosis	Drugs in use	Highest school level completed	Family mental disease
1	F	23				High school	No
2	M	34				Middle school	No
3	M	28				High school	No
4	M	23				High school	No
5	M	27				High school	No
6	F	40				Elementary school	No
7	F	26				High school	No
8	M	28				High school	No

#### Apparatus and Procedures

A laptop (Sony^®^ Vaio^®^, Brazil) connected to a 15" monitor (Bematech^®^, USA) was used for data collection. All training and test tasks were created and run using the E-Prime 3.0 software (Psychology Software Tools Inc^®^, USA). The test stimuli consisted of the Brentano version of the MLI (Figure 1B). The main axis of the illusion had 20 cm of length (approximately 20 visual degrees) with one fixed arrowhead at each end pointing in the same direction (the direction for each trial was randomly assigned). A third arrowhead pointing in the opposite direction was placed on the main axis between the other two arrowheads. The tip of this central arrowhead was used as the spatial reference to divide the main axis into two segments. All arrowheads subtended a 90° angle formed by two 4-cm long lines. For each experimental session, the participants sat comfortably in front of the screen, kept at a 50-cm distance from their eyes. Each trial started with the presentation of a blank screen for 2 s followed by a tone buzzer. After that, the Brentano MLI stimulus was presented in the center of the screen. On each trial, the central arrowhead would randomly appear at any point on the main axis, between the two other arrowheads. The participants were asked to slide the central arrowhead along the main axis, using the keyboard arrows, so that its tip would divide the main axis into two segments of equal length. The illusory effect of the arrowheads was determined by the distance between the actual center of the axis (i.e., 20 cm) and the participant’s estimation. When this had been completed, the participant would press “space bar” and a new trial would start immediately. Each participant was submitted to a total of 20 trials.

At the end of the illusion testing session, the patients (but not control participants) were subjected to a brief version of the PANSS (Positive and Negative Syndrome Scale). The scale was administered by a trained psychiatrist and took approximately 30 min.

### Statistical Analysis

For the PSE stage, the difference in length between the pair of lines with and without arrowheads was compared using a paired *t*-test. For the Test stage, the percentage of correct responses on the four-session sequence within each treatment was averaged. Mean values were then analyzed using a two-way analysis of variance (ANOVA) with repeated measures on treatment (vehicle and ketamine) and stimuli pair type (“illusion pair” and “neutral pair”). *Post hoc* comparisons were performed whenever appropriate using Tukey’s test. For the human experimental data, schizophrenic and control performance was compared using unpaired *t*-test. Data are presented as the mean PSE percentage or mean percentage of correct answers ± standard error of the mean (SEM). The significance level for all tests was set at *p* < 0.05.

## Results

**Figure 2:**
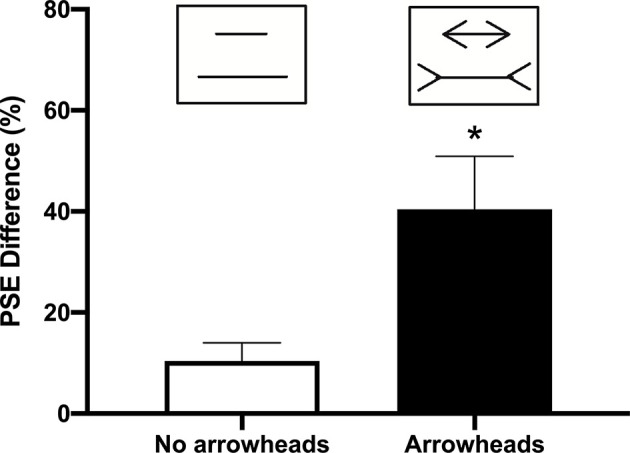
Difference in the point of subjective equality (PSE) with and without arrowheads. Bars indicate the average of the length difference between the two lines obtained when determining the PSE with and without arrowheads (mean + SEM; *n* = 5; *p < 0.05).

**Figure 3:**
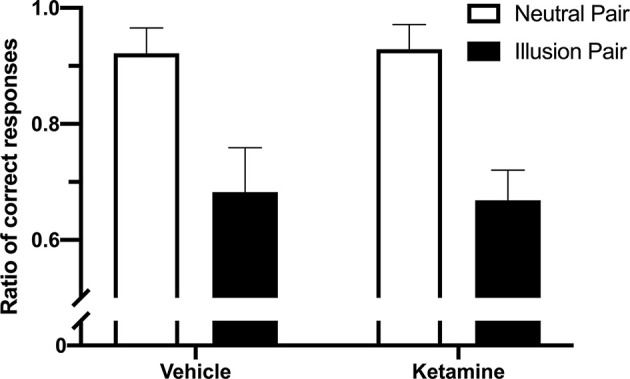
Effects of ketamine administration on the percentage of correct responses between “illusion” and “neutral” pairs (mean + SEM; *n* = 4).

In Experiment 1, the PSE difference for the lines without arrowheads (10 ± 3.75 cm) was significantly smaller than those with arrowheads (40.4 ± 10.53 cm; *t*_(4)_ = 6.175; *p* = 0.0035; Figure 2). For the percentage of correct responses on the ketamine test, the repeated measures ANOVA indicated a significant difference between the illusion conditions (“neutral pairs” vs. “illusion pairs”; *F*_(1,12)_ = 80.96; *p* < 0.0001; Figure 3). The number of correct responses recorded for the “illusion pairs” was significantly lower than that for the “neutral pairs”. On the other hand, there were no significant between-treatment differences (*F*_(1,12)_ = 0.017; *p* = 0.898) or factor interaction (*F*_(1,12)_ = 0.138; *p* = 0.716; Figure 3).

**Figure 4:**
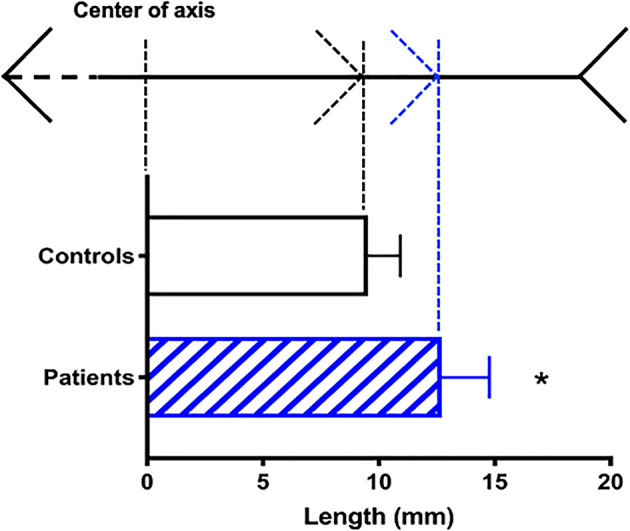
Susceptibility to the Müller-Lyer Illusion among schizophrenic patients (blue) and controls (black). On the top: reference points in the illusion axis (not to scale). Average deviation in mm (mean + SEM) from the actual center of the axis (*n* = 16; *p < 0.05).

In Experiment 2, schizophrenic patients misplaced the center of the axis by a significantly larger margin than did the controls (p < 0.05; *t*_(14)_ = 3.751; Figure 4). The patients’ estimations were off by 12.01 ± 0.72 mm (approx. 1.44° ± 0.08° visual angle), whereas the error margin for controls was 9.53 ± 0.49 mm (approx. 1.09° ± 0.06°; Figure 4). No correlation was found between MLI performance and PANSS main scores (positive: *p* = 0.92; negative: *p* = 0.45; cognitive: *p* = 0.37; data not shown).

## Discussion

The present study corroborated previous findings that capuchin monkeys are susceptible to the MLI and showed that chronic schizophrenic patients are more susceptible than controls. The effects of the MLI on the monkey’s length judgment in Experiment 1 were similar to those reported in previous studies with capuchins (Suganuma et al., 2007; Jacobsen et al., 2017) and rhesus monkeys (Tudusciuc and Nieder, 2010). In the present experimental setup, the monkeys were not restrained during training or testing to reduce stress. This approach precluded the exact determination of the actual visual angle subtended by the stimuli on the screen. Nonetheless, given the average distance, the animals were from the screen during the task, we can estimate the judgment deviation (in this case, the difference between PSE and the actual length of the line) to be approximately 1.2°, similar to the values presented by Tudusciuc and Nieder (2010) in two rhesus monkeys.

In our experiment with the human participants, the task required sliding the central arrowhead in a Brentano version of the MLI. Interestingly, the average judgment deviation (i.e., the strength of the illusion) found in this modified task was that of 1.09° of the visual angle in healthy participants. This result is a little lower than that reported for the original version of the MLI in humans (1.19°: Tudusciuc and Nieder, 2010). The absence of time limits for completing each trial in our experiment may explain the difference across studies. In our task, participants took a variable length of time to slide the arrowhead, depending on its starting position in each trial, but generally, it took longer than the general time limits used on forced alternative designs. As such, it has been reported that the strength of the Brentano may decrease if participants are allowed to inspect the stimulus (Predebon, 1998), but the duration of the inspection itself was not relevant. Despite this difference, the present task allowed for a faster and more precise (as confirmed by the low data dispersion in both groups) method to evaluate the judgment deviation in this task.

There has been some controversy regarding the sensitivity to MLI in schizophrenia patients. To our knowledge, the majority of reports have found robust changes compared to controls. These include increased susceptibility and/or impaired length estimation (Weckowicz and Witney, 1960; Capozzoli and Marsh, 1994; Rund et al., 1994; Parnas et al., 2001; Kantrowitz et al., 2009; Shoshina et al., 2011, 2014; Tolmacheva et al., 2018) or even reduced sensitivity depending on the progression of the disease (Parnas et al., 2001). Some reports, however, failed to find any significant differences (Tam et al., 1998; Grzeczkowski et al., 2018). Also, Kaliuzhna et al. (2019) argued that schizophrenia patients do not show perception abnormalities in early levels of visual hierarchy that supports a general impairment to visual illusions. Although, their results may corroborate the null findings for some visual illusions they did not test MLI specifically and, therefore, should not refute the several significant findings in the scientific literature on this illusion. The few conflicting results more likely stem from the methodological differences employed, as pointed out by King et al. (2017). Most studies used the standard illusion scheme with either a size-judgment or a forced-choice task which have been criticized in this context (Skottun and Skoyles, 2014). In this sense, the present approach, i.e., the sliding Brentano version, sought to provide a quantitative and sensitive measure of the participants’ PSE which yields a high intra-subject reproducibility. We acknowledge that our results with patients are preliminary and based on a small sample size. It corroborates the results of most articles with MLI but care must be taken when comparing with other studies.

Another source of discrepancy among previous studies is sample heterogeneity and/or medication profile. Few studies have attempted to compare across schizophrenia subtypes/chronicity, let alone pharmacological treatment. One such study (Parnas et al., 2001) found that prodromal and first episode patients were less sensitive than controls in a forced-choice task with the Brentano version of MLI. On the other hand, chronic patients did not show a statistically higher susceptibility to the illusion. Here, the strength of the illusion was indeed higher for chronic patients compared to controls. The precision yielded by our sliding task may account for this difference, although Parnas et al. (2001) did not present a full statistical description in their results. Furthermore, we found that the MLI deviation was not correlated to the PANSS scores. Once again, this is hardly surprising given the low variability in the MLI results, in stark contrast to the psychometric 3-axis information evaluated by the PANSS scale. Alternatively, the lack of correlation might also reflect the absence of sensory/perception dimensions in the PANSS test. Therefore, it would be helpful to test a larger and more diverse sample of schizophrenia subtypes in the sliding Brentano task to characterize a possible range of illusion strength within this disorder and correlate it with the quantitative symptoms across the spectrum.

In a previous study, we found that NMDA receptor blockade by the antagonist MK-801 decreased the strength of the illusion in capuchin monkeys (Jacobsen et al., 2017). This effect seems to be in line with results from prodromal and first episode patients (Parnas et al., 2001). Here, we attempted an alternative strategy by blocking the receptor with ketamine. Surprisingly, the 4-day ketamine administration regimen failed to induce changes in performance as had been detected with MK-801. It is believed that NMDA blockage is the main pharmacological mechanism underlying the psychotic effects of ketamine and MK-801 (Lisman et al., 2008). Although both drugs share a common NMDA blocking site, ketamine shows a relatively lower affinity than MK-801 (Bresink et al., 1995). It also has a broader binding profile and is known to exert a wider range of effects, including anesthesia at higher doses. These effects may be induced by its moderate to strong interactions with cholinergic (Moaddel et al., 2013), dopaminergic (Kapur and Seeman, 2002), and opioid sites (Hirota et al., 1999), although the precise mechanisms are not well clarified.

One possible caveat to our results would be the relatively low dose chosen for ketamine. Previous studies with capuchin monkeys reported increase salivation, dystonia, reduced locomotor activity, and impaired reaction to stimuli in doses ranging from 2.5 to 5.0 mg/kg (Shigii and Casey, 1999, 2001). Taffe et al. (2002) found that 1.0 mg/kg, but not 0.3 mg/kg, of ketamine, disrupted cognitive performance in macaques. A prior pilot testing in our lab also showed mild ataxic movements at 1.0 mg/kg (data not shown). Since the present task required accurate precision for stimuli selection, we opted for a dose of 0.3 mg/kg. It is noteworthy that the dose used by Jacobsen et al. (2017) for MK-801 was also very low (5.6 μg/kg) and yet it was able to improve performance in this task. A low dose of ketamine also decreases the chance of a significant activation of aminergic or opioid systems. The discrepancies between ketamine and MK-801 effects in the MLI task are, therefore, more likely to stem from their different affinities with the NMDA receptor.

Regardless of the actual mechanism, currently available data seems to suggest that MK-801 may be the only effective drug to affect illusion strength in the present protocol. This may be somewhat problematic for this model since it restricts the range of effects that can be tested. Increasing MK-801 doses may easily induce ataxia which prevents MLI task execution. Therefore, even if higher doses could increase, rather than decrease, illusion strength, the current protocol would not be suitable to detect it. As sample sizes in monkey experiments are generally small due to ethical limitations, a continuous ketamine infusion protocol, similar to that used in human subjects (Lahti et al., 2001), may enable the individual calibration of ketamine blood levels and ensure a stable, and possibly, a dose-dependent effect throughout the task duration.

Even though the sliding Brentano task may be too challenging to use with nonhuman primates, the forced-choice MLI task yields comparable results and may still be used for antipsychotic drug screening. It may also be tested in other models for schizophrenia, such as neonatal hippocampal lesion or PCP administration. On the other hand, given the range of sensory/perceptual alterations in patients and the lack of perceptual indices for diagnostical purposes, the task with Brentano is a promising tool in both research and clinical settings as it may be easily deployed on laptops or tablets for patient testing. In light of the results gathered so far on the MLI, further investigation is warranted on different schizophrenia subtypes and their immediate relatives, as well as a better refinement of nonhuman primate protocols.

## Data Availability Statement

The raw data supporting the conclusions of this article will be made available by the authors, without undue reservation.

## Ethics Statement

The studies involving human participants were reviewed and approved by University of Brasília Research Ethics Committee (CAAE 96510318.3.0000.0030). The patients/participants provided their written informed consent to participate in this study. The animal study was reviewed and approved by Animal Ethics Committee of the University of Brasilia (46077/2014).

## Author Contributions

RM conceived the study, designed the experiment and wrote the first draft of the manuscript. RS was responsible for experimental setup, data collection, and analysis in Experiment 1. PC-C participated in experimental setup and data collection in Experiment 1. AC was responsible for experimental setup, data collection and analysis in Experiment 2. FS participated in experimental setup and data collection in Experiment 2. MB and FC participated in the experimental design planning, data analysis, and manuscript drafting. All authors contributed to the article and approved the submitted version.

## Conflict of Interest

The authors declare that the research was conducted in the absence of any commercial or financial relationships that could be construed as a potential conflict of interest.
